# Cerebellar-Induced Aphasia After Stroke: Evidence for the “Linguistic Cerebellum”

**DOI:** 10.1007/s12311-024-01658-1

**Published:** 2024-01-20

**Authors:** Djaina Satoer, Peter J. Koudstaal, Evy Visch-Brink, Ruben S. van der Giessen

**Affiliations:** 1 Department of Neurosurgery, Erasmus MC University Medical Center, Dr. Molewaterplein 40, room Na-2106, 3015 GD Rotterdam, The Netherlands; 2 Department of Neurology, Erasmus MC University Medical Center, Rotterdam, The Netherlands

**Keywords:** Cerebellar stroke, Aphasia, Linguistic cerebellum, Cerebellar cognitive affective syndrome, Neurolinguistic tests, Diagnostic Instrument for Mild Aphasia

## Abstract

The cerebellum is traditionally known to subserve motor functions. However, for several decades, the concept of the “cerebellar cognitive affective syndrome” has evolved. Studies in healthy participants and patients have confirmed the cerebellar role in language. The exact involvement of the cerebellum regarding *cerebellar aphasia* remains uncertain. We included 43 cerebellar stroke patients who were tested at 3 months post-onset with the Boston Naming Test (BNT), the Token Test (TT), and the Diagnostic Instrument for Mild Aphasia (DIMA). Lesion side (left/right) and volume (cm^3^) were investigated. Patients significantly deviated on the following: BNT (*p*<0.001), TT (*p*<0.05), DIMA subtests: sentences repetition (*p*=0.001), semantic odd-picture-out (*p*<0.05), sentence completion (*p*<0.05) without an effect of lesion location (left/right) or volume (cm^3^) (p>0.05). Our clinical study confirms a non-lateralized cerebellar aphasia post-stroke, characterized by impairments in word retrieval, phonology, semantics, and syntax resembling cerebral-induced aphasia. The integral cerebellum appears to interact with eloquent cortico-subcortical language areas.

## Introduction

The cerebellum is traditionally known to play a role in motor control [1]. However, for about three decades, the concept of “cerebellar neurocognition” has evolved. In seminal work from Schmahmann and Sherman [2], a consistent pattern of cognitive and affective deficits was described in patients with focal cerebellar lesions and was coined as the “cerebellar cognitive affective syndrome” (CCAS). This condition was characterized by impairments in executive functions (e.g., set-shifting, planning, abstract reasoning), visuo-spatial cognition, personality changes (e.g., flattening or blunting of affect, disinhibited or inappropriate behavior), and a variety of linguistic deficits (e.g., dysprosodia, agrammatism, decreased verbal fluency, and mild anomia). Next, several case studies of patients with a cerebellar lesion and (experimental) functional neuroimaging studies in healthy participants contributed to knowledge about cerebellar function in the context of neurocognition and CCAS [3]. Most patient group studies were heterogeneous (e.g., tumor, degenerative, stroke). Only one study specifically included isolated cerebellar stroke patients, but post-onset times varied from acute to chronic stage (up to more than a year) [4]. It is therefore difficult to draw solid conclusions about the role of the cerebellum with respect to characteristics of CCAS. Generally, the effect of lesions is expected to be more subtle than cortical lesions since the cerebellum acts as a parallel system to fine-tune motor behavior rather than generating direct motor output.

Although considerable evidence has been gathered with regard to neurocognitive functions and the cerebellum, the exact role of the so-called linguistic cerebellum is still under debate. Experimental, neurophysiological, and neuroimaging studies have confirmed that the cerebellum is involved in several linguistic functions, such as grammar processing, verbal fluency [5, 6], and reading and writing [7]. Cerebellar-induced aphasia has also been described in several case studies. Mariën et al. [8] reported a 73-year-old, right-handed patient who suffered from word finding difficulties, lack of spontaneous speech initiation, expressive and receptive grammatical difficulties, and reading and writing deficits which led to the term cerebellar-induced aphasia. Single photon emission computed tomography (SPECT) studies in this patient showed a significant hypoperfusion not only in the right cerebellum, but also in the prefrontal language region of the left dominant hemisphere. Follow-up data showed paralleled patterns between perfusion change and neurolinguistic results confirming the role of cerebellum in language [9]. In addition, a lateralized involvement of lateral posterior cerebellar regions (including lobules VI and Crus I/II) was found in anatomo-clinical studies of patients with focal cerebellar lesions and linguistic impairments. In non-clinical populations, a relation between the cerebellum and the cerebral cortex with regard to language functioning has also been found ([10]; and for meta analyses, see [11]). This points to crossed cerebello-cerebral connections between the cortical language network and the cerebellum. In patient populations, which were often heterogeneous or only case studies, aphasia-like symptoms were not consistently present after cerebellar disease. Increased reaction times in a verb generation task were found, whereas performance on Aachener Aphasia subtests was intact or without aphasic characteristics in spontaneous speech [12, 13]. It has been suggested that cerebellar-induced aphasia is transient and most prominent in the acute phase ([7, 14]. Possibly, aphasia tests, designed for “classical” stroke population, are not sensitive enough to capture (mild) language deficits in cerebellar stroke patients. Correct diagnostics is of crucial importance, as not only moderate to severe aphasia but also mild aphasia can have detrimental effects on patients’ quality of life [15].

In this study, part of a large prospective study on Cognitive Deficits after Cerebellar Stroke (CODECS) [16], we investigated language functioning in a homogenous patient group with an isolated vascular cerebellar lesion, confirmed by imaging, by means of an extensive neurolinguistic protocol including tests designed for mild aphasia at 3 months post stroke onset. We hypothesized that a new test for mild aphasia would identify impairments, whereas standard aphasia tests would be less sensitive to detect disturbances. Finally, we expected that language impairments would be more pronounced in patients with right cerebellar stroke.

## Materials and Methods

We included patients with focal cerebellar lesions admitted to the Department of Neurology at the Erasmus MC University Medical Center Rotterdam between April 2015 and April 2019. Exclusion criteria consisted of extra-cerebellar lesions, pre-existent neurocognitive or psychiatric disorders, and age younger than 18 years. Approval by the local Medical Ethics Committee was obtained (MEC-2013-462). All patients gave written informed consent and were given a standard neurological evaluation. After 3 months post-stroke, each patient underwent structural neuroimaging by means of computed tomography (CT) and/or magnetic resonance imaging (MRI) scan to confirm an isolated cerebellar lesion. All patients were investigated by means of a neurolinguistic assessment at 3 months. A standard neuropsychological protocol and the International Cooperative Ataxia Rating Scale (ICARS) were used to quantify the cerebellar ataxia as a measure of motor severity (for details see van der Giessen et al., [16].

### Language and Cognitive Measures

We used the shortened Token Test [17] to determine the presence and severity of aphasia. To assess word retrieval, the Boston Naming Test (BNT) [18] was administered. The BNT consists of 60 black and white objects ordered in descending level of word frequency. Subtests at the linguistic levels phonology, semantics, and grammar from the recently developed Dutch Diagnostic Instrument for Mild Aphasia (DIMA) [19] were administered: verbal repetition (words, compounds, non-words, sentences), semantic odd-picture-out, sentence completion (see Table 1). Error analyses on deviant language tests were conducted. Errors were calculated into percentages and divided into the following categories: semantic, irrelevant (i.e., not semantically related) or phonematic paraphasia, hesitation, slow response, self-correction, anomia, superordinate, circumlocution, neologism, perseveration, repetition/omission/insertion of words or no reaction.

**Table 1:** Test protocol

Test	Cognitive abilities	Description
Language		
Shortened Token Test (TT) [17]	Language comprehension; severity of language disorder	Pointing to and manipulating geometric forms on verbal commands
Boston Naming Test (BNT) [20]	Naming (word finding)	Naming 60 pictures, presented in order of word frequency and word difficulty
Diagnostic Instrument for Mild Aphasia (DIMA) [19]	Phonology: repetition of words, non-words, and sentences Semantics: semantic odd-picture out under time pressure Syntax: sentence completion	Repeating existing and non-existing words, sentences increasing in complexity (e.g., gorilla, anáto, Every Friday we eat freshly made curry soup) Naming objects/animals that do not semantically fit in a series of three (e.g., snake, cat, dog) Sentence completion with a word and constituent (e.g., I wash my hands with …; Every day ….)
Attention and executive functions		
Trail Making Test (TMT) A [21]	TMTA: visuomotor speed, attention	Connecting numbers placed randomly in ascending order as rapidly as possible (TMT A)

Nonverbal cognitive abilities were assessed using the Trail Making Test A (TMT-A) [22]. In the TMT-A, the patient connects numbers (1–25) in an ascending order on a paper sheet. The score consists of the time in seconds it takes to finish. Visuo-perceptual speed underlies performance on the TMT-A (scores from TMT-B and BA are not taken into account in this paper).

### Statistical Analysis

Based on published normative data, raw test scores of the patients were transformed into *z*-values to compare the performance of patients and healthy adults (when possible corrected for age, education, and sex). We investigated whether patients’ mean test *z*-scores differed from the normal population, using a one-sample *t*-test with 0 (the mean score of the normative group) as test value. To minimize the number of statistical comparisons, only tests of which the mean performance deviated from normal population were selected for further comparisons, such as the influence of lesion location (left/right cerebellum) on language scores with a univariate analysis of variance. Pearson rank correlations were made between scores on the Token Test, deviating DIMA subtests harboring a speed component and TMT-A. Lesion location and volume were extracted from the CT/MRI and lesion volume was calculated in squared centimeters by a neurologist (RG).

## Results

Forty-three patients were included in the study. Demographic characteristics are summarized in Table 2. Eighteen (41.9%) patients were men. The majority was right-handed (81.4%). The average age was 62 years (SD 15; range 22–92); mean education was 12 years (SD 3.46; range 6–22). The majority of the lesion was located in the right cerebellum (58.1%) with a mean volume of 15.87 cm^3^.

**Table 2:** Demographic and clinical characteristics

Patients *N*=43	
Sex (male)	18 (41.9%)
Age—mean; range	62.65; SD 15.53
Education (years)	12.41; SD 3.46
Handedness (right)	35 (81.4%)
Localization stroke	
*Cerebellum*	
Right	15 (34.9%)
Left	25 (58.1%)
Midline	3 (7%)
Stroke volume in cm^3^ mean (0–71.6)	15.87

### Language Measures

**Fig. 1:**
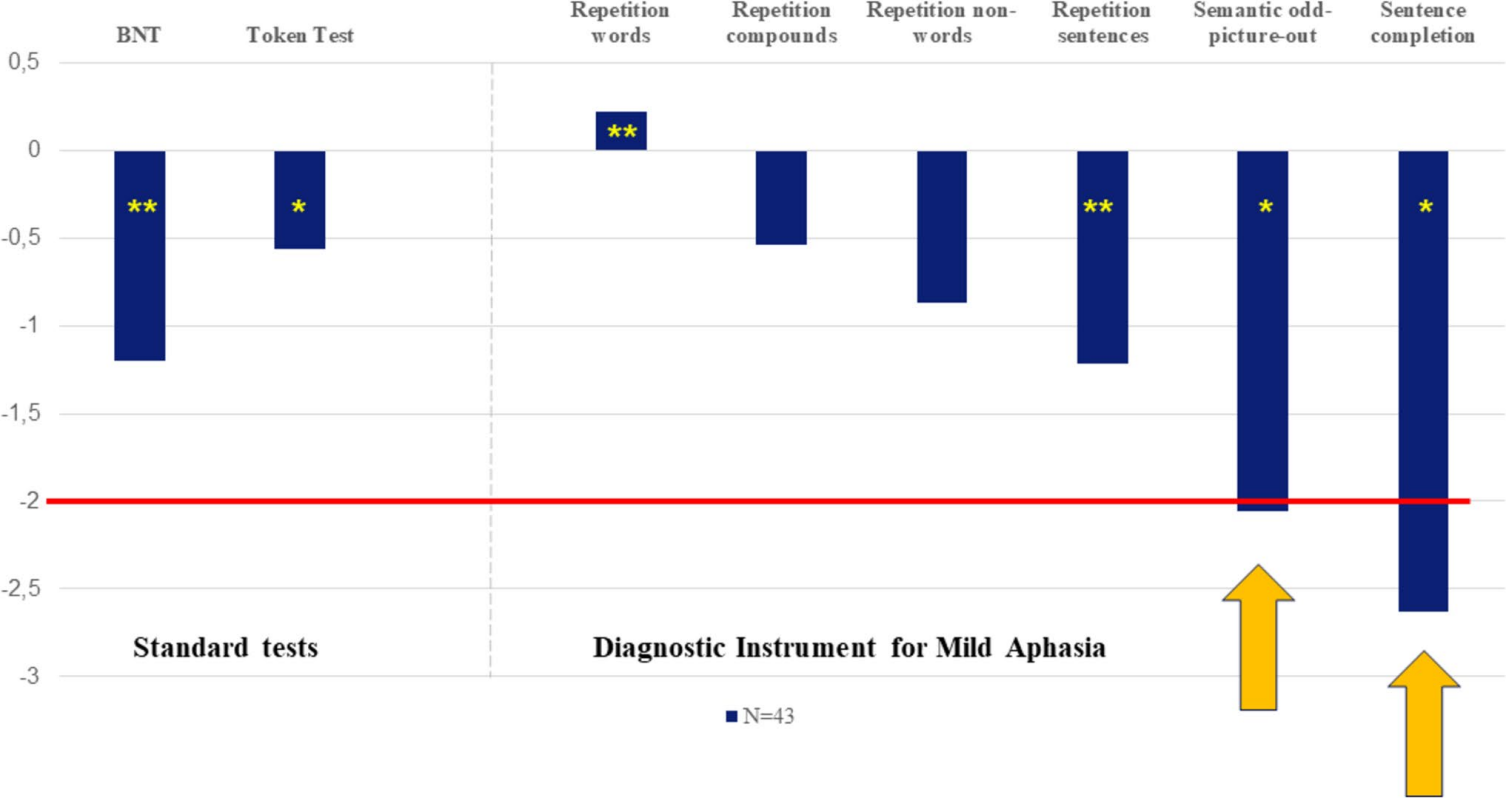
Mean *z*-scores standard language tests and DIMA cerebellar stroke patients. One-sample *t*-test, *p<0.05, **p=0.001. The bold (red) line (and see also orange arrows) indicates the threshold for a clinical impairment (*z*-score = ≤ −2)

**Fig. 2:**
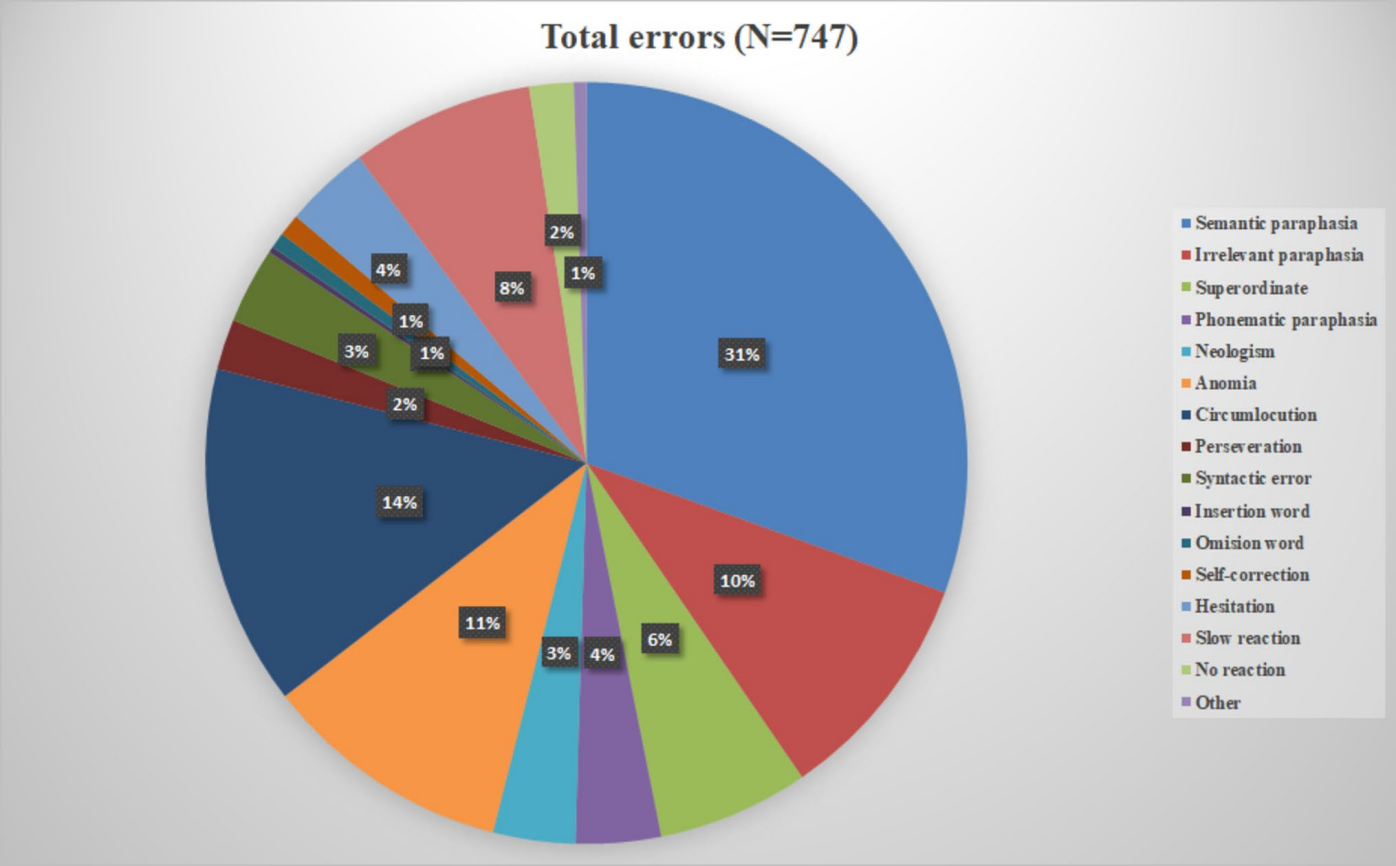
Error analysis in percentages (total errors in tests: BNT, sentence repetition, semantic odd-picture-out, sentence completion)

Patients’ mean scores were statistically worse compared to normal population on the BNT (*z*-score = –1.20, *t*= –4.339, *p*<0.001) and the Token Test (*z*-score= –0.56, *t*=–2.475, *p*<0.05). No clinically impaired scores were found (*z*= ≤ −2). For the DIMA subtests, statistical deviations between patients and normal population were found in the following: repetitions of words (*z*-score= 0.22, *t*=3.057, *p*<0.001), repetition of sentences (*z*-score= −0.68, *t*=−3.421, *p*=0.001), semantic odd-picture-out (*z*-score= −0.64, *t*=−2.635, *p*<0.05), and sentence completion (*z*-score= −0.64, *t*=−2.113, *p*<0.05) (see Fig. 1 and Table 3 for an overview). The subtests semantic odd-picture-out and sentence completion were also clinically impaired (*z*= ≤ −2). There was a significant moderate positive correlation between scores on repetition of sentences, sentence completion, and the Token Test (Pearson *r*=0.615, Pearson *r*=0.472, *p*<0.01 resp.), and a significant weak positive correlation was found between scores on semantic odd-picture out and the Token Test (Pearson *r*=0.384, *p*<0.05). Qualitative error analyses on deviant language tests revealed various patterns; see below in Table 4 error percentages per test and total errors in percentages in Fig. 2.

**Table 3:** Overview results one-sample test, language scores of cerebellar stroke patients

	Raw score/max score (SD)	Z-score (SD)	*T*	Df	Sig. (2-tailed)	Mean difference	95% confidence interval of the difference	
							Lower	Upper
Boston Naming Test	48.74 / 60 (8.44)	−1.20 (1.81)	−4.339	42	.000	−1.20279	−1.7622	−.6433
Token Test	33.29 / 36 (2.93)	−0.56 (1.47)	−2.475	41	.018	−.56024	−1.0174	−.1031
Repetition words	9.78 / 10 (0.68)	0.22 (0)	3.057	42	.000	.22000	.2200	.2200
Repetition compounds	9.64 / 10 (0.96)	−0.54 (2.35)	−.055	42	.956	−.00907	−.3414	.3233
Repetition non-words	8.93 / 10 (1.49)	−0.87 (1.58)	−1.415	42	.165	−.24860	−.6032	.1060
Repetition sentences	8.78 / 10 (1.54)	−1.22 (1.60)	−3.421	42	.001	−.67744	−1.0771	−.2778
Semantic odd-picture out	4.37 / 5 (0.99)	−2.06* (2.28)	−2.635	42	.012	−.63767	−1.1261	−.1492
Sentence completion	9.26 / 10 (1.18)	−2.63* (3.88)	−2.113	42	.041	−.64047	−1.2521	−.0289

*Clinical impairment (*z*= ≤ −2)

**Table 4:** Qualitative error analysis per deviating language tests (in %)

Error type	BNT (*N*=559)	Sentence repetition (*N*=85)	Sem odd-pic-out (*N*=44)	Sentence completion (*N*=59)
Semantic paraphasia	*36.5%*		*54.6%*^1^	
Irrelevant paraphasia	10.2%	*20%*		
Superordinate	8.9%			
Phonematic paraphasia	1.4%	*22.3%*		
Neologism	4.7%			
Anomia	*14.1%*			
Circumlocution	*19.3%*			
Perseveration	0.4%	9.4%		*10.1%*
Syntactic error				*40.7%*
Insertion word		2.4%		
Omission word		5.9%		
Self-correction	3.9%	7.1%	2.2%	
Hesitation		*31.8%*		
Slow reaction (> 4 s)			*27.3%*	*40.7%*
No reaction		1.1%	*15.9%*	8.5%
Other	0.6%			

^1^In this case, an incorrect odd picture out was named. As all items belong to the same main category with one subcategory as the odd one out, f.i. three animals of which two are domestic animals and 1 is not, this error can be considered a semantic paraphasia. The three most frequent errors *per test* are in italics

### Influence of Lesion Location and Non-linguistic Performance Versus Language Tests

No effect of cerebellar lesion location (left/right) was observed on the deviating language tests (*p*>0.05). Also lesion volume did not correlate with language performance (*p*>0.05). To investigate possible influence of non-linguistic slowness on semantic odd-picture-out and sentence completion as high rates of slow reactions (see Table 4) were observed, Pearson rank correlations were conducted with TMT-A. No correlations were found between TMT-A and semantic odd-picture out and sentence completion (Pearson *r*=0.211, *p*=0.511; Pearson *r*=−0.191, p=0.552).

## Discussion

We investigated neurolinguistic functioning in a large homogeneous group of patients with isolated cerebellar stroke. Disturbances were found not only in standard aphasia tests (BNT and Token Test) but also in subtests from the Diagnostic Instrument for Mild Aphasia (repetition of sentences, semantic odd-picture-out, and sentence completion), which clearly confirms a role in language functioning for the cerebellum. Our patient group was consistently tested with a neurolinguistic protocol at 3 months post-onset. These findings show that cerebellar-induced aphasia is not just transiently present in the acute phase as commonly described in the literature [7, 14].

Different aspects of language in both production and comprehension were found to be impaired compared to normal population, including word retrieval (object naming), a test for aphasia severity/comprehension (Token Test), phonological production/working memory (repetition of sentences), rapid semantic selection/word retrieval (semantic odd-picture-out), and spontaneous speech in context (sentence completion). Apart from DIMA, also standard aphasia tests were statistically deviant, which suggests that the language profile of cerebellar stroke patients resembles aphasic disturbances caused by a cerebral lesion albeit to a less severe extent.

As for the standard aphasia tests, the Token Test is well-known to determine the presence and severity of aphasia [17]. It could be debated whether in our study the severity was mild, as the Token Test score was statistically deviant but not clinically impaired (cutoff *z*=−2). Deviating DIMA subtests correlated moderately with the Token Test, indicating that higher scores on DIMA subtests were associated with better performance on the Token Test. The Boston Naming Test was also sensitive for statistical deviations in the cerebellar patient group indicating problems with word retrieval. Our qualitative analysis showed that the most frequently occurring errors concerned semantic paraphasias, circumlocutions, and anomias. These are typical error types in aphasic population (f.i. anomic aphasia). A naming deficit was described earlier in cerebellar patients. Baillieux et al. [23] investigated a heterogeneous cerebellar group (tumor, arteriovenous malformation, infarction) of 18 patients of which 22% presented with a naming deficit. Fabbro et al. [24] also described problems in lexical retrieval in four right-handed cerebellar tumor patients. After surgery, only two patients partially recovered. In vascular case studies, (isolated) word retrieval deficits were also found [8, 25–27].

The test-battery for mild disorders, DIMA, also detected deficits in production subtests in the linguistic levels phonology, semantics, and syntax. At the level of phonology, repetition of sentences was impaired. Most commonly produced errors concerned hesitations, phonological, and irrelevant paraphasias which are (partially) different from the commonly reported distorted articulation, motor speech planning (apraxia of speech), or prosody in the context of ataxic dysarthria or verbal apraxia in patients with cerebellar lesions [28, 29] (see also [16], in which a negative correlation between ICAR score and language tests was found). The test items in our subtest are constructed to be phonologically complex as they involve phonemic similarities (tongue twisters), e.g., *de Griek ontdekte vier nietjes in de band van zijn fiets* (The Greek discovered four staples in the tire of his bike). It was found earlier that this so-called phonological similarity effect causes difficulties in cerebellar (degenerative and focal) lesions and in children with cerebellar tumor removal. Memory for phonologically similar words was worse than for phonologically dissimilar words and that this could have been caused by a deficit in phonological store [30, 31]. In addition, a defect in articulatory rehearsal in light of the *forward output model*, usually to explain motor function (e.g., prediction of limb state during movement trajectory), may have played a role. It is postulated that this forward output model can also control articulatory trajectory, that is, the prediction of the sequence of articulatory movements needed to rehearse verbal information formed during the initial encoding of the verbal stimuli [32]. The rapid engagement of the phonological loop via such a mechanism could increase the likelihood that the phonological store is refreshed before it has had chance to fade. Apart from computing the correct phonological information into lexical items and articulation, repetition of sentences also makes use of working memory as words need to be temporarily stored in a buffer or phonological loop [33] (see also [34] in which the articulatory side of the “Baddeley-Hitch model” is argued to be premature and less related to articulation). Some studies have demonstrated deviations in working memory, attested with impairments in tests such as digit or letter span forward and backwards [25]. Another test in which working memory also is involved is verbal fluency. Leggio et al. [35] found that in cerebellar patients, phonological fluency performance was worse than semantic fluency performance, due to the absence of reference to meaning in the phonological fluency test; hence, a stimulus has to be maintained in the working memory buffer (produce as many words within 1 m starting with letter “F”). In our larger dataset with the same patients from Van der Giessen et al. [16], verbal fluency (semantic and letter) was also impaired.

The second subtest from DIMA that deviated was the semantic odd-picture out. This is a test in which both semantic judgement and word retrieval have to be executed under time pressure. Apart from semantic fluency, semantic processing has received little attention in the field of cerebellar lesions [7]. Not only in this DIMA subtest, but also in the total number of errors, semantic paraphasias (incorrect selection semantically related item) were most frequently produced, followed by slow and no reactions in the semantic odd-picture-out. Semantic abilities (e.g., decision, discrimination, association) have been described to be impaired in focal cerebellar lesions [36], and to be involved in the right cerebellum in healthy participants with neuroimaging studies [37, 38]. It is remarkable that no reactions and semantic paraphasias were also observed in the BNT, a test for word-retrieval, confirming problems in word retrieval and activation of the wrong lexical item. Slow reactions were exceeding the time span of 4 s per item and seemed to be language specific, thus independent from a more general non-linguistic cognitive processing speed (TMT-A) as no correlations between these tests were found.

The final sensitive DIMA subtest was sentence completion. Sentence completion is a test for spontaneous speech in context and known to be sensitive to detect dynamic aphasia [39]. A loss of spontaneous speech initiation was earlier seen in the reported case study with a cerebellar lesion by Mariën et al. [8]. Most frequently occurring errors in our subtest concerned syntactic errors, slow reactions followed by repetition of a word. A high rate of syntactic errors in the sentence completion test shows that there could be some (mild) form of syntactic production deficit (e.g., errors in word order, inflections) in accordance with several case illustrations [8, 40–42]. Silveri et al. [41] found that a syntactic deficit in a cerebellar patient was most prone to morphology (omission of auxiliaries or inflection errors). They argued that the impairment was not considered to affect syntactic competence but is targeted to the online application of syntactic rules to correctly assign grammatical morphemes. This could also be due a problem in a reduction of cognitive resources [43]. However, in our dataset, the lack of a correlation with a more general non-linguistic cognitive processing speed (TMT-A) contradicts this statement.

In contrast to our expectations, we did not find a “lateralized linguistic cerebellum” as no significant differences were found between left and right cerebellar stroke patients. There are other studies who also found that left-sided or bilateral cerebellar lesions can lead to language disruption [40]. Based on this study, it indeed seems that the integral cerebellum interacts with eloquent cortico-subcortical areas related to specific linguistic functions, such as word retrieval in the inferior frontal gyrus, the middle inferior and anterior middle temporal gyrus, and the supramarginal gyrus and the inferior longitudinal fasciculus. Semantics in the posterior superior temporal gyrus and inferior fronto-occipital fasciculus, and spontaneous speech in context (initiation) in the supplementary motor area, the angular gyrus, the frontal aslant tract ([39, 44]. It is also possible that a larger sample size in combination with neuroimaging techniques such as resting-state functional connectivity is needed to demonstrate specific crossed cerebro-cerebellar circuits for language [45]

In addition, a more detailed division according to the functional linguistic topography by Stoodley and Schmahmann [46] could have been more sensitive to detect differences in language performance: that is the anterior lobe, parts of the medial lobule IV, lobule VIII of the posterior lobe, and the interpositus nuclei form the sensorimotor cerebellum, where the cognitive cerebellum consists of lobule VII, parts of lobule VI, Crus I and II, and the ventral part of the dentate nuclei. Lesion studies have confirmed a role for the dentate nucleus within the deep cerebellar nuclei (DCN) in language. Cerebellar mutism was described in a patient following two posterior fossa tumor operations; the first resection the right DCN was partially persevered with intact speech, whereas the second resection concerned bilateral involvement of DCN resulting in mutism [47]. Involvement of the DCN on language performance could be investigated in the future in a larger dataset. On the other hand, it can be argued whether this detailed cerebellar division is practical in clinical use.

Future analyses of the cerebellum serving as a *forward output model*, usually to explain motor function (i.e., dysmetria), should be expanded to language function or even other cognitive functions (“dysmetria of thought”) and the discussion as to whether or to which extent the cerebellum acts as an error-based learning mechanism in “cognitive cerebro-cerebellar loops” [48]. In the context of language, some psycholinguistic experiments in healthy participants have confirmed the role of the cerebellum in linguistic/semantic prediction [49, 50] in parallel with the more general prediction during both motor control. This may underlie the high occurrence of semantic errors in total and the lowest performance in sentence completion in which a part of the sentence needs to be “predicted.” This more general line of thought may contradict evidence in favor of an aphasia with an actual “loss of linguistic function.” More clinical experiments in cerebellar aphasia including detailed error analyses must be conducted in order to support this viewpoint.

This prospective study clearly confirms the role of the cerebellum in language function at several modalities (production and comprehension) and linguistic levels (word retrieval, phonology, semantics, and syntax) and that patients indeed suffer from a(n) (mild) aphasia. This is relevant information for clinical practice as language deficits can negatively influence patients’ social and professional life. We therefore recommend to administer a (short) neurolinguistic protocol in cerebellar stroke patients in the first months post-onset with preferably complex tests at different modalities and levels (e.g., tests under time pressure). The application of (suitable) language therapy should be investigated further. Other cerebellar diseases need to be investigated in a comparable uniform way in order to draw more solid conclusions about cerebellar-induced aphasia. The modalities reading and writing were not taken into account, but should be studied in the future as a relation between dyslexia and the cerebellum is known [28]. It seems in this study that other cognitive functions, such as non-linguistic processing speed, do not influence language functioning. However, the underlying mechanism of cerebellar-induced aphasia still remains to be elucidated.

## Data Availability

Not applicable.
